# Effects of NMES-Guided Scapular Retraction Exercise Program in Amateur Female Handball Players with Scapular Dyskinesis Without Shoulder Pain: A Randomized Controlled Clinical Trial

**DOI:** 10.3390/jcm14155567

**Published:** 2025-08-07

**Authors:** Luis Espejo-Antúnez, Javier Gutiérrez-Coronado, Carlos Fernández-Morales, Manuel Albornoz-Cabello, Luis Fernando Prato, María de los Ángeles Cardero-Durán

**Affiliations:** 1 Department of Medical-Surgical Therapy, Faculty of Medicine and Health Sciences, University of Extremadura, 06006 Badajoz, Spain; luisea@unex.es (L.E.-A.); jgutierrezc20@gmail.com (J.G.-C.); mcarderod@unex.es (M.d.l.Á.C.-D.); 2 Department of Physiotherapy, University of Seville, 41009 Seville, Spain; malbornoz@us.es; 3 Lakeshore Bone and Joint Institute, 3691 Willowcreek Rd, Portage, IN 46368, USA; coachprato@hotmail.com

**Keywords:** electric stimulation therapy, exercise therapy, handball, scapula, shoulder joint/pathology

## Abstract

**Objective:** This study aimed to evaluate the effect of simultaneously combining therapeutic scapular retraction exercise with and without Neuromuscular Electrical Stimulation (NMES) in amateur female handball players with scapular dyskinesis. **Methods:** In a randomized, single-blind, controlled trial, the sample (n = 34) was randomized into two groups (Group 1 (n = 17) and Group 2 (n = 17)). The intervention consisted of applying a supervised scapular retraction exercise (SRE) program alone or combined with NMES for 4 weeks (2 ss/week). Scapular Static Positioning Assessment parameters (upper and lower horizontal distance of the scapula from the spine (mm)), internal rotation range of motion (degrees), and external rotation strength (newtons and BW%) were measured. **Results:** A significant interaction was found to favor the group that received the supervised SRE program + NMES (Group 1) in upper horizontal distance (F_1,30_ = 30.93 [*p* < 0.000]; *d* = 0.65); lower horizontal distance (F_1,30_ = 12.79 [*p* = 0.001]; *d* = 0.72); ER Strength (N) (F_1,30_ = 19.58 [*p* < 0.000] *d* = 0.71); and ER Strength (BW%) (F_1,30_ = 16.84 [*p* < 0.000]) *d* = 0.69), which was statistically significant (*p* ≤ 0.001 for *p* < 0.05). In the analysis for treatment benefit, the number needed to treat (NNT) was 2 for upper scapular positioning and 4 for external rotation strength. **Conclusions:** NMES improves the Scapular Static Positioning and ER Strength when combined with an SRE program in amateur female handball players diagnosed with scapular dyskinesis, with clinically relevant effects. These findings, while promising, are based on a small sample and should be confirmed in larger studies.

## 1. Introduction

Shoulder injury and pain have a high incidence among the so-called “overhead sports athletes” [1]. Among them is handball, which is characterized by a high number of throws and frequent physical contact [2,3]. Handball is a sport that requires a combination of strength, speed, resistance, and power [4]. Most shoulder injuries in handball players are related to the repetitive throwing movements [5,6], occurring most frequently at the end of the match or during training [7]. Among younger players (14–18 years), there is an overuse injury rate of 1.4/1000 h of play. Among elite athletes this rate increases, reaching up to 3.7/1000 h in training and 20.3/1000 h in matches [5]. Clarsen et al. [2] reported that 24% of the players have dominant-shoulder problems at some point during the season, with a weekly prevalence of 12%.

Shoulder disorders in overhead athletes such as handball players are highly associated with scapular dyskinesis (SD) [8,9]. SD is defined as altered scapular position and movement. In 2018, a meta-analysis showed that asymptomatic athletes with scapular dyskinesis have a 43% greater risk of developing further shoulder pain than those without SD [8]. Specifically, female handball players have higher injury incidence rates than male players (26% vs. 20%) [3,10]. Most scapular-related injuries in throwing athletes can be traced to loss of control of normal resting scapular position and dynamic scapular motion. Some studies have suggested that shoulder complex adaptations, such as excessive posterior displacement of the inferior angle prominence, insufficient posterior tipping of the scapula, altered scapular muscle performance, or movement relative to the thoracic cage [6,9,11,12], may be related to the presence of alterations in scapular control and movement without pain [13].

In this sense, previous studies have emphasized the role of any type of therapeutic exercise (e.g., scapular muscle strengthening, scapular stabilization exercise, stretching) in restoring normal scapular kinematics [14,15,16]. Recently, Moghadam et al. [15] in a systematic review study confirmed the beneficial effects of stabilization exercises on scapular motion and position. However, Kamonseki et al. [16] found no superior improvements for scapular movement training compared with standardized exercises in terms of scapular biomechanics or behavioral and clinical outcomes, and the most effective type of exercise remains currently unclear. Given this, Colson et al. [17] analyzed the effects of the combination of different types of exercise with Neuromuscular Electrical Stimulation (NMES) in subjects with scapulo-humeral dystrophy. These authors reported positive effects in the strength and function of the scapula humeral musculature after applying therapeutic exercise combined with NMES for 5 months. Traditionally, NMES has been used to maximize muscle activation and force production capacity. The underlying mechanisms that explain its effects rely on its ability to recruit motor units through the depolarization of motor neurons, thereby eliciting skeletal muscle contractions of substantial intensity [18]. In addition, Baldwin et al. [19] obtained significant improvements in range of motion (RoM), pain, and shoulder disability after applying, in survivors of head and neck cancer, a rehabilitation program based on bilateral therapeutic exercises consisting of scapular retraction and elevation with NMES for 6 weeks.

Despite the feasibility, safety, and effectiveness shown in different populations, there are no studies using NMES and analyzing its effects on the performance of handball players with shoulder pain [17,19]. Specifically, to the authors’ knowledge, there are no studies that analyze the effectiveness of a specific program in female handball players at recreational and amateur levels. Furthermore, the use of NMES as a possible feedback aid in sports pre-habilitation (transition from conscious muscle control to strength training) [20] of handball players [21] has not been considered in other studies.

Taking into account the clinical and sporting performance implications of SD in the shoulder complex [2,22] of recreational amateur handball players without shoulder pain [23], the aim of this study was to evaluate whether adding NMES to a scapular-focused exercise protocol is more effective than the scapular-focused protocol alone on static scapular positioning, glenohumeral internal rotation RoM (IRRoM), and external rotation strength in amateur female handball players with SD.

## 2. Materials and Methods

### 2.1. Study Desgin

The present study is a randomized, single-blind, controlled trial. This study was supervised by the Bioethics Committee of the University of Extremadura, with ethics approval number 107/2021, approved on 29 September 2021, registered in ClinicalTrials.gov (NCT04432441) on 12 June 2020, and in accordance with the Declaration of Helsinki. We followed CONSORT statements, and all the subjects agreed to take part in the study and signed the informed consent.

### 2.2. Participants

The potentially eligible sample (n = 82) consisted of female handball players who practiced handball at least 6 h/week at recreational (without economic remuneration) or amateur levels. Participants were recruited from the Barcelona Sants handball club. Recruitment was carried out through direct coordination with the coaching staff, and informative sessions were held at the club’s own facilities. The inclusion criteria were (i) female handball players; (ii) age between 18 and 40 years; (iii) federated club membership that allows them in their weekly practice to participate in recreational matches or small local championships during the weekends [23]. A qualitative examination for determining the presence or absence of SD was applied by two physiotherapists, independent of the study, with more than 8 years of experience in the diagnosis of SD. They identified the presence or absence of SD by observing the static position at rest or the dynamic motion upon arm motion using the scapular dyskinesis test:

(a) Static scapular assessment: The test was assessed in a neutral and standing position as proposed Sayaca et al. [24]. SD was accepted as positive if the difference between the upper horizontal distance and lower horizontal distance was >1.5 cm.
(b) Visual-based palpation classification method for SD according to Huang et al. [25]: SD was classified as a single abnormal scapular pattern [inferior angle (pattern I), medial border (pattern II), superior border of scapula prominence or abnormal scapulohumeral rhythm (pattern III)], a mixture of the above abnormal scapular patterns, or a normal pattern (pattern IV). The assessment of SD was evaluated as the subjects performed bilateral arm raising/lowering movements with a weighted load (1.5 kg) in the scapular plane (5 repetitions). This method has shown moderate-to-substantial inter-rater reliability [25].
(c) Test battery according to the McClure scale [26]: This measurement represents a reliable and feasible method for clinical examination of overhead athletes. Each test movement (flexion and abduction) was rated as follows:

Normal: Both test motions are rated as normal, or 1 motion is rated as normal, and the other as having a subtle abnormality.
Subtle abnormality: Both flexion and abduction are rated as having subtle abnormalities.
Obvious abnormality: Either flexion or abduction is rated as having obvious abnormality.
Exclusion criteria: players with presence of instability shown by a positive apprehension test, sulcus sign, and/or positive anterior drawer [27] at the time of recruitment or within the last 12 months; (ii) history of recurrent shoulder subluxation [28]; (iii) drug consumption with potential effects on balance and/or postural control in the 24 h prior to the initial assessment; (iv) having participated in an exercise-based preventive program or having had shoulder complex surgery in the last 12 months [26,29].

### 2.3. Sample Size Calculation

Sample size calculation was based on the following: taking into account a one-tailed hypothesis, an alpha value of 0.05, a desired power of 80%, and a large effect size with Eta square (η^2^) = 0.27, 30 participants were required in total. The G * Power software, 3.1.9.2 version, was used (Düsseldorf, Germany).

**Figure 1:**
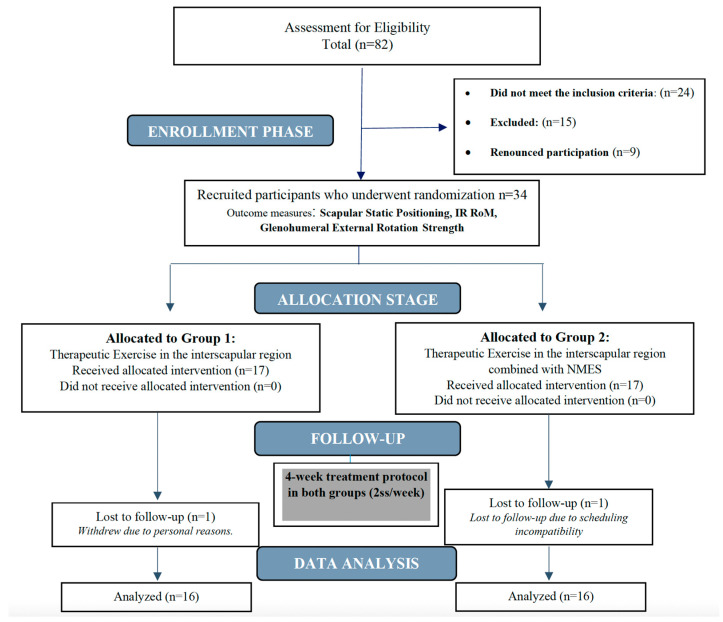
Flowchart of participant recruitment.

Finally, the sample consisted of 34 female players (18–35 years) (Figure 1).

### 2.4. Randomization

Participants were randomly (using block randomization, 1:1) allocated into two groups: Group 1 (n = 16 participants) and Group 2 (n = 16 participants). An evaluator researcher (J.G.-C.), aware of the design of the study, was responsible for the enrollment and group assignment. Participants from both groups were assessed at the beginning of the session (baseline evaluation) and after 4 weeks (post-intervention). Both the statistician and the physiotherapist carrying out the intervention were blinded. Outcome assessors remained blinded to group allocation throughout both data collection and analysis.

### 2.5. Procedures

All subjects were assessed on the dominant upper limb. The assessments followed the same order at baseline and after the intervention. To avoid being influenced, the participants of both groups were evaluated in separate rooms, at the same temperature, in order to maintain the same environmental conditions between subjects. All assessments and procedures were carried out at the training facilities of the Barcelona Sants handball club.

#### Outcomes Measures

Scapular Static Positioning (SSP) Assessment: The upper horizontal distance and lower horizontal distance were used, as proposed by Larsen et al. [30]. Both scapular clinical tests quantify by tape measure (mm) the distance between the upper edge of the base of the scapular spine and the corresponding spinous process of T3 vertebra and the distance between the lower edge of the scapula and the spinous process of T7 vertebra, respectively. Both measures have shown good psychometric properties in the assessment of scapular position [ICC (95% CI) = 0.80 (0.62–0.89); SEM: 0.67] and function [ICC (95% CI) = 0.71 (0.46–0.85); SEM: 0.76], respectively [30].

Glenohumeral IR RoM: The test was performed prior to training so that soft tissue elasticity and muscle fatigue would not affect the joint angle RoM. The participants were placed in the supine position on the measurement table. The subject’s shoulder was abducted to 90º, and the elbow was flexed to 90°, as per Cieminski et al. [28]. A two-arm goniometer (Lafayette^®^, Lafayette, IN, USA) was used. This measurement has shown good psychometric properties [ICC (95% CI) = 0.95 (0.88–0.98); SEM: 2.16] in amateur handball players [23]. Three measurements were taken, selecting the one with the highest range as in previous studies [2,4,29].

**Figure 2:**
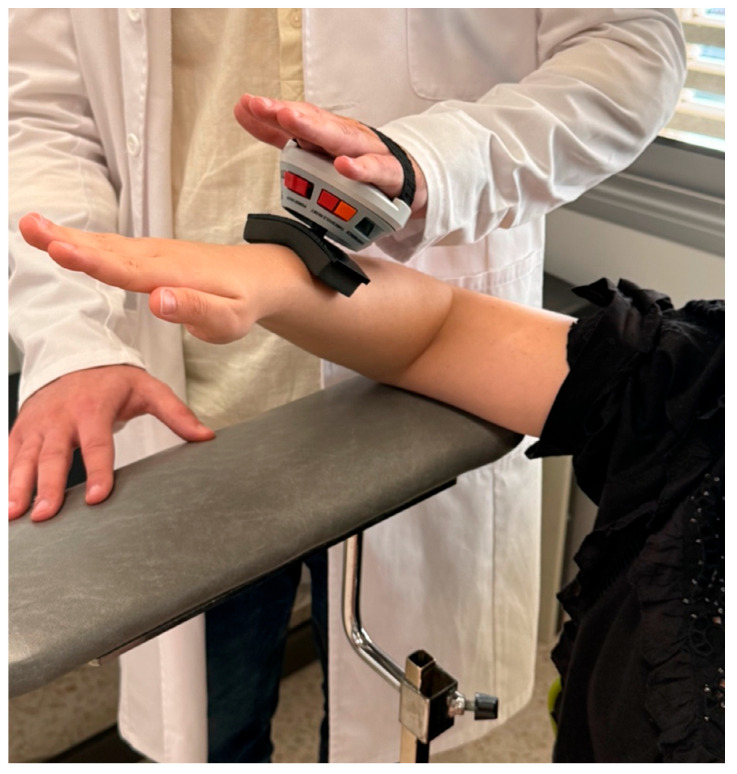
Evaluation of isometric external rotation strength using a hand-held dynamometer.

Glenohumeral External Rotation Strength (ERS): The maximal isometric voluntary strength of the external rotator muscles was measured using a hand-held dynamometer (microFET2; Hoggan Health Industries^®^, Salt Lake City, UT, USA). The instrument was calibrated before each measurement. An adaptation of the procedure described by Guney et al. [29] was used for the measurement. The tester placed the dynamometer on the back of the participant’s wrist and applied force to the wrist, gradually increasing in 3 to 5 s (Figure 2). Participants were instructed to resist the applied force (break test) [23]. At the point at which the participant could no longer resist the force (maximal isometric contraction) and the arm began to move, the test was terminated, and the force was recorded. Each participant was tested 3 times. The patient was placed in a standing position with the back and arm to be analyzed leaning against the wall, with 90° of shoulder abduction, 90° of elbow flexion, and 0° of rotation. Measurements were made through an arc of 80° of ER, with respect to the horizontal. Each subject was familiarized with the testing procedure at 50%, 75%, and 90% of their perceived maximum effort. The individual was then instructed to perform maximal efforts with a 15 s recovery between each ER effort and a 30 s rest between each set. Three trials were performed, and the best of the 3 values was used for analysis. Subjects received standardized verbal cues from 1 investigator (M.d.l.Á.C.-D.) and no visual feedback. The ICC and SEM for ER peak torque were 0.77 and 1.4 Nm.

The contralateral arm remained alongside the body. Verbal and manual assistance was provided to stabilize the scapula before the test, with no external fixation used during the procedure. Once in position, the participants were instructed to push into external rotation (dynamometer handle attached to the hand) with increasing force up to maximal voluntary force and hold for 3 s in order to avoid injury and minimize the risk of dynamic contractions. Three trials were performed, with 30 s rest in between each other in order to enhance maximum voluntary contraction stability and avoid muscle fatigue [31]. The units of measurement used were both newtons and kilograms. Kilograms were standardized by the weight of each individual, multiplied by 100, and expressed as percentage of body weight (%BW) as proposed by Vigolvino et al. [23]. This measurement procedure has shown high intra-examiner reliability [ICC (95% CI) = 0.87 (0.72–0.95); SEM: 0.37/0.73].

### 2.6. Intervention

The interventions took place in a rehabilitation scenario with full activity with supplemental conditioning for all participants. The positions used to achieve scapular position and motion in shoulder function were retraction in cocking, wheelbarrow with push plus/bench to push-up, and controlled protraction [32].

**Figure 3:**
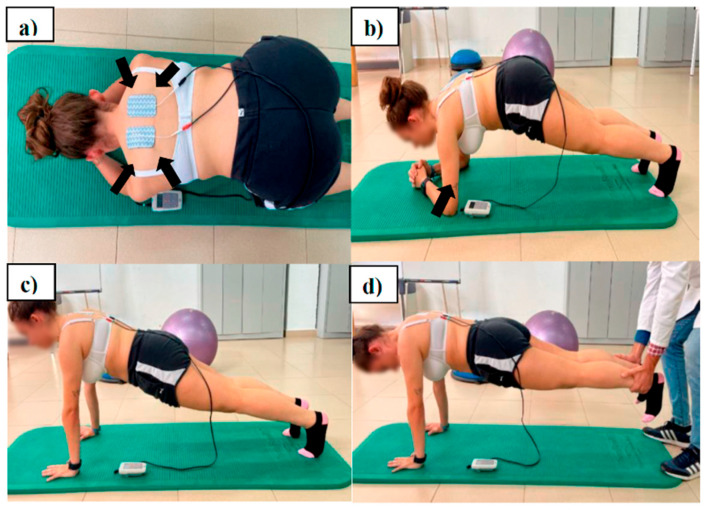
Supervised scapular retraction exercise program combined with NMES (final position). (**a**) V-to-Y scapular retraction and external rotation. (**b**) Protraction exercise I: prone hip bridge with active scapular control. (**c**) Protraction exercise II: push-up transition with protraction. (**d**) Wheelbarrow: partner-assisted scapular stabilization exercise.

Group 1 (n = 16) received an intervention protocol using the SRE program. The program consisted of a four-part sequence of isometric prone push-up and wheelbarrow (partner exercise) with push plus/bench to push-up, it being an optimal position for scapular strengthening/control exercises [33,34] (Figure 3). First, the participants were placed in the prone position with their arms flexed and abducted to 45–50° with their torso. Elbows were placed beneath the shoulder. The forearms and hands were placed apart, with the thumbs pointing up. Subjects first retracted and depressed their scapula, making sure that the upper trapezius were relaxed. Maintaining retraction of the scapula, subjects then isometrically externally rotated their arms, keeping 45–50° of shoulder abduction for the entire exercise.

Secondly, subjects were positioned in a prone hip bridge with forearms and toes supporting the body on the floor and with their arms flexed and abducted to 120° with their torso. Then, they pushed-up 1–2 cm, protracting the scapula, but actively attempting to prevent scapular winging.

Thirdly, participants pushed their upper body up with their elbows extended, pulled the shoulders forward, and changed the position to a push-up position and back to the starting position again.

Finally, the fourth exercise consisted of making the wheelbarrow. The starting position was the same as that for the second exercise, but the partner supported the ankles. Stabilization of the scapula and forward head control throughout the exercise routine was emphasized during instruction.

Three sets of 8 repetitions were performed for the first exercise, with 1 min rest between each set. The duration of the exercise was 8 s followed by 10 s of rest after each repetition. The second and third exercises were performed in 2 sets of 6 repetitions each. The last exercise (the wheelbarrow) was performed in 2 sets of 15–30 s according to tolerance. The SRE program lasted 8 sessions (2 sessions/week) over a period of 4 weeks. The total time per session was 12 min.

Group 2 (n = 16) received the same intervention combined simultaneously with the use of NMES (biphasic symmetrical pulse, bipolar mode, 50 Hz frequency, pulse duration 300 µs at 1:1 ratio) (S82^®^; Enraf-Nonius BV, Rotterdam, The Netherlands) based on established recommendations [35]. The self-adhesive electrodes (8 × 5 cm^2^ Pals Platinum © type, Axelgaard Manufacturing Co. Ltd., Fallbrook, CA, USA) were placed on the middle and lower trapezius muscle bilaterally, with the positive electrode placed on the dominant limb. The blinded investigator (M.d.l.Á.C.-D.) used the superointernal and inferior angle of the scapula as a reference (Figure 3a,b).

The overcurrent program consisted of 8 s of work (ON phase) and 8 s of rest (OFF phase). The overcurrent program in the ON phase had a rise time of 2 s, a hold time of 4 s, and a fall time of 2 s. The intensity of the electric current was increased to the maximum perceived tolerance, achieving a clear contraction without producing fatigue. The instructions were “When you start to feel the electric current with perception of muscle contraction, it is time to start the exercise. You must hold the position during the 4 s that lasts the current pulse”.

The rest of the instructions were the same as those given for Group 1. The number of sessions, weekly frequency, and periodicity were identical for both groups. The interventions were carried out by a physiotherapist with 15 years of experience in using exercise therapy and NMES. To avoid the risk of contamination between participants, the two groups were assessed in separate rooms. None of the participants in the study had received theoretical or practical training about the procedure applied before the intervention. The interventions were carried out in compliance with the recommendations of the Consensus on Exercise Reporting Template (CERT) statement [36] and the Template for Intervention Description and Replication (TIDieR) checklist [37].

### 2.7. Statistical Analysis

The statistical analysis was carried out using PASW Advanced Statistics (SPSS Inc, Chicago, IL, USA), version 24.0. Firstly, the normal distribution of variables was verified by the Shapiro–Wilk test, after a descriptive analysis. The Levene test was used to assess the homogeneity of variances. Linearity was assessed by bivariate dispersion graphics of residual values observed from the expected values. Comparisons between groups were made for demographic and clinical data of reference using Student’s t test for continuous variables and Pearson’s chi-square test for categorical variables.

Differences in measurements were detected by analysis of variance of repeated measures (ANOVA) 2 × 2, mixed analysis of variance (ANOVA) to evaluate group × time interactions, including the effect of time (baseline, four weeks after treatment) as an intra-subjects’ factor and group effects (Group 1 vs. Group 2) as inter-subjects’ factors. Furthermore, the effect size was calculated through the Cohen’s d coefficient. A value above 0.8 was considered high, around 0.5 was moderate, and lower than 0.2 was considered low.

To assess treatment benefit, the relative risk (RR), absolute risk reduction (ARR), relative risk reduction (RRR), and number needed to treat (NNT) were calculated for the primary outcomes, along with their 95% confidence intervals. These metrics were based on the proportion of participants in each group who achieved a clinically meaningful improvement, as defined by predefined cut-off values [30].

Additionally, bivariate correlations (Pearson or Spearman, as appropriate) were performed to examine potential associations between baseline sociodemographic characteristics and outcomes showing statistically significant between-group differences. The significance level was established at *p* < 0.05.

## 3. Results

A total of 32 subjects, with ages between 18 and 35, were included. In total, 28 of the shoulders treated were the right ones (87.5%) while the remaining 4 were the left ones (12.5%). The majority of the sample showed a single obvious abnormality (pattern I and II)**.** The demographic characteristics and baseline outcomes measures are shown in Table 1. Statistically significant differences were not found at the inter-group level for any of the variables (*p* > 0.05 for all).

Although the outcomes measures have been proved to be an accurate tool to assess the status of the scapular dyskinesis, the reliability results for the present study were as follows: ICC and Standard Error Measurement (SEM) were calculated for upper scapular distance = 0.771 [0.512–0.884], SEM: 2.82; lower scapular distance = 0.793 [0.573–0.898], SEM: 1.71; ER Strength (Kg) = 0.733 [0.435–0.865], SEM: 0.09; and ER Strength (%BW) = 0.802 [0.584–0.901], SEM: 0.19.

Table 2 includes baseline and final measurements, as well as the between-groups and intra-group mean differences. Statistically significant differences were found to favor the SRE + NMES group in upper horizontal distance (F_1,30_ = 30.93 [*p* < 0.000]; *d* = 0.65); lower horizontal distance (F_1,30_ = 12.79 [*p* = 0.001]; *d* = 0.72); and ER Strength (F_1,30_ = 19.58 [*p* < 0.000] *d* = 0.71) and ER Strength (BW%) (F_1,30_ = 16.84 [*p* < 0.000]) *d* = 0.69).

The analysis for treatment benefit showed that 11 participants (68.8%) in the intervention group improved beyond the minimal detectable change for upper scapular distance (≥18.4 mm) [28], whereas only 1 participant (6.3%) in the control group achieved this level of improvement. The NNT was 2 (95% CI: 1.18–6.22), with an RR of 11.00 (95% CI: 1.60–75.50), an ARR of 0.625 (95% CI: 0.161–0.847), and an RRR of 0.992 (95% CI: 0.376–0.987). In contrast, no statistically significant effects were observed for the lower horizontal distance, and the calculated effect measures did not indicate meaningful between-group differences.

Similarly, 15 participants (93.8%) in the intervention group improved beyond the MDC in ER Strength (≥3.88 N), while 11 participants (68.8%) in the control group also met this threshold. The NNT was 4 (95% CI: 1.98–∞), with an RR of 0.20 (95% CI: 0.03 to 1.53), an absolute risk reduction (ARR) of 0.25 (95% CI: −0.01 to 0.51), and a relative risk reduction (RRR) of 0.80 (95% CI: −0.53 to 0.97). These results did not reach statistical significance but indicate a clinically relevant trend in favor of the intervention.

## 4. Discussion

The aim was to evaluate the effects of combining an NMES-guided scapular retraction exercise program in amateur handball players diagnosed with painless SD.

Rehabilitation approaches should be reconsidered where enhancing motor control becomes the primary focus rather than increasing strength [38]. To date, the improvements achieved on muscle strength by exercising the scapular stabilizers that restore muscle balance in overhead sports athletes are well known [33,39]. However, the variety in types of exercise and the lack of scientific evidence on recommendations according to specific sports disciplines has made it difficult to determine which exercises are effective in achieving both goals (motor control and strength). The reported results may suggest that NMES is an excellent facilitator of scapular retraction, thereby improving scapular position and muscle recruitment.

On the other hand, these findings are consistent with the recommendations of the American Medical Society for Sports Medicine on the importance of rehabilitation programs to monitor risk factors in adolescent athletes [40]. Recently, Asker et al. [34] found that adolescent handball players who undertook a preventive shoulder exercise program reduced the absolute risk of injury by 11%, with the number needed to treat at 9. Our results support the statements made by Clarsen et al. [2] on the need to incorporate exercise in injury prevention programs, to improve total rotational motion, external rotational strength, and scapular position.

Regarding SSP, statistically significant improvements were found in both groups (*p* ≤ 0.05). Group 1 showed results that differed partially from those obtained by Lynch et al. [41] after applying a scapular stabilization exercise program in elite swimmers. These differences could be explained by the following reasons: (i) recorded measures of scapular position vary between studies; (ii) the technical gesture of swimmers and handball players is performed in different conditions, which could influence the effects of the proposed exercises; (iii) while the inclusion criteria of the present study required the existence of SD, the criteria indicated by Lynch et al. [41] were focused on the forward head posture and shoulders.

Group 2 also showed significant increases, with moderate and large effect sizes at the upper horizontal distance (mean differences: 23.1 mm, *d* = 0.65) and lower horizontal distance (mean differences: 19 mm; *d* = 0.72), respectively. These results improved the minimum detectable change (MDC) for the upper horizontal distance established by Larsen et al. [30] by 18.4 mm, which is close to the 21.1 mm indicated by these authors for the lower horizontal distance.

Statistically significant differences were also observed between groups in favor of the Group 2. From a neurophysiological point of view, NMES induced a gain in strength, achieving current intensities at the levels that elicited maximum muscle contraction without causing unacceptable discomfort [42]. Placement of the electrodes for selective activation of inhibited or weaker muscles (middle trapezius, lower trapezius, and serratus anterior) with minimal activation of hyperactive muscles (upper trapezius) during the controlled exercise of scapular retraction and shoulder protraction could restore the muscular balance, improving the orientation of the scapula, as proposed by Huang et al. [39]. These results indicate a possible influence of the flow of the electrical current on conscious scapular control. Future studies are necessary to know the combined effect of NMES and therapeutic exercise on the scapular orientation in overhead athletes with and without SD.

Neither group showed significant changes in IR RoM after the intervention nor were there significant changes between groups. Likewise, the changes found were lower than the MDC (3.05°) indicated by Vigolvino et al. [23]. Although the glenohumeral IR deficit (GIRD) is one of the main risk factors of glenohumeral joint damage in overhead sports athletes [40], previous studies have also shown that athletes who play handball at the amateur level do not present GIRD, showing no significant differences between dominant and non-dominant shoulder in players at this competitive level [23]. The absence of changes should be interpreted with caution, as it may reflect a potential protective effect of the intervention by minimizing the deterioration of this parameter described in overhead athletes. Future studies should analyze both dominant and non-dominant sides, with longer follow-up periods.

On the other hand, the absence of deficits in IR RoM is consistent with the lack of significant association shown by Clarsen et al. [2] in handball players with slight or obvious dyskinesis (OR 95% CI: 0.64 (0.14–1.19); *p* = 0.19). The IR RoM values obtained in both groups for the throwing arm differ partially from recent studies performed in different overhead sports disciplines [2,28,42]. Our results showed higher ranges in both groups than those reported by Lee et al. [35] in elite young baseball athletes without GIRD (41.98 ± 15.29 vs. Group 1: 47.2 ± 3.68 vs. Group 2: 45.2 ± 6.83); furthermore, they were lower than those reported by Guney et al. [29] on the dominant shoulders of teenage basketball and volleyball players (52.11 ± 8.92). These differences could be due to possible tissue adaptations arising from the level of competition (elite vs. amateur) shown in the study by Lee et al. [35] and from the average age and experience in sports practice when comparing our results with those shown by Guney et al. [29].

Statistically significant intra- and between-group improvements were also observed in ER Strength. These results are consistent with those reported in a recent systematic review on the positive impact of isometric strength training in female handball players [43]. The between-group differences were in favor of Group 2, with large effect sizes (ER Strength in newtons: *d* = 0.71; ER Strength in BW%: *d* = 0.69) (Table 2). According to Cools et al. [13], the benefits could be explained by the stimulation of the lower and middle trapezius during the isometric glenohumeral external rotation movement. The superior benefits after applying NMES have been related to enhanced activation of motor units as well as an increase in the muscle cross-sectional area, which may lead to greater structural, metabolic, and neural adaptations [44].

### 4.1. Clinical Impact of Interventions, as Assessed by Number Needed to Treat

Beyond statistical significance, the clinical relevance of our findings was supported by the impact estimates derived from dichotomized responder analyses. For upper scapular positioning (Upper HD), the NNT was 2 (95% CI: 1.18–6.22), indicating that treating two players with NMES-guided exercises would lead to one additional meaningful improvement compared with control. The risk ratio was 11.00 (95% CI: 1.60–75.50), with an ARR of 0.625 and an RRR of 0.992. In ER Strength, although confidence intervals crossed the null value, the NNT remained favorable (4; 95% CI: 1.98–∞), with an RR of 0.20 and an ARR of 0.25. These trends align with the motor control emphasis highlighted by Huang et al. [39] and support the combined utility of NMES and isometric training in amateur handball athletes. Furthermore, these estimates are comparable to recent preventive programs in adolescent athletes, such as the work by Asker et al. [34], who reported an NNT of 9 in reducing shoulder injuries. From a clinical perspective, such a magnitude of benefit with relatively small sample sizes may justify integrating NMES into sport-specific prevention and conditioning protocols.

### 4.2. Clinical Implications

The role of strength training programs performed on the shoulder to optimize the sports performance (throwing speed, muscle power, …) of young female handball players has been hypothesized [45]. However, NMES-guided exercise to improve scapular position in addition to strength has not been investigated to date. The interventions could be useful in other sports disciplines that work in the overhead position and involve SD (pattern I and II mainly). Taking into account that combining exercise with NMES seems to be effective in subjects with SD without pain, the implementation in prevention programs could be considered a relevant practical implication by sports players who need to improve scapular position, adapting the placement of the electrodes to the inhibited muscles where muscle activation could improve their sporting performance.

### 4.3. Limitations

Some potential limitations should be acknowledged. Firstly, the study included participants from a single specific sport discipline, which limits the generalizability of the results to other overhead athletes. Moreover, all the participants were asymptomatic, so the applicability of these findings to individuals with shoulder pain remains uncertain. Secondly, alternative NMES protocols were not considered. The existing heterogeneity in stimulation parameters restricts the discussion regarding the optimal dosing for scapular dyskinesis. Thirdly, the duration of the intervention (4 weeks) may be considered short compared to previous NMES studies lasting 6–12 weeks. No follow-up assessment was conducted, so the long-term retention of effects remains unknown. Finally, no sham NMES was applied in the control group.

## 5. Conclusions

NMES-guided scapular retraction exercises applied for 4 weeks appear to be more effective than the verbally guided exercise program for Scapular Static Positioning and for muscle strength at external rotation in amateur female handball players with SD.

## Figures and Tables

**Table 1:** Baseline characteristics of participants in the study groups.

	Total Sample (n = 32)	Group 1 (n = 16)	Group 2 (n = 16)	*p* Value *
Mean age (years)	23 (3.23)	24 (3.75)	23 (2.69)	0.593
Height (cm)	167 (4.31)	166 (4.66)	167 (4.01)	0.470
Weight (kg)	60.8 (8.01)	61.0 (9.01)	60.7 (7.18)	0.914
Body mass index	21.6 (2.25)	21.8 (2.51)	21.4 (2.03)	0.623
Hours of sport/week	7 (1.01)	7 (1.05)	7 (0.95)	0.194
Throwing shoulder (right/left)	28/4	14/2	14/2	1
Scapular dyskinesis test				
Pattern I (%) (subtle/obvious abnormality)	14 (43.7) Subtle: 3 Obvious: 11	8 (50)	6 (37.5)	
Pattern II (%) (subtle/obvious abnormality)	11 (34.4) Subtle: 2 Obvious: 9	5 (31.2)	6 (37.5)	
Pattern III (%) (subtle/obvious abnormality)	2 (6.2) Obvious: 2	1 (6.2)	1 (6.2)	
Pattern IV (%) (subtle/obvious abnormality)	5 (15.6) Subtle: 1 Obvious: 4	2 (12.5)	3 (18.7)	
Upper horizontal distance (mm)	86.7 (13.41)	87.2 (12.37)	86.2 (14.77)	0.847
Lower horizontal distance (mm)	89.1 (10.77)	89.7 (11.89)	88.4 (9.87)	0.749
IR RoM (°)	40.2 (5.49)	41.2 (3.68)	39.2 (6.83)	0.296
ER strength (N)	45.29 (7.47)	44.15 (7.75)	46.11 (7.26)	0.777
ER strength (BW%)	7.75 (1.28)	7.67 (1.46)	7.75 (1.16)	0.739

Data are reported as mean (SD); SRE: scapular retraction exercise; NMES: Neuromuscular Electrical Stimulation; RoM: range of movement; (°): degrees. * Between-groups statistical significance (one-factor ANOVA).

**Table 2:** Baseline, post-intervention, and mean score changes in shoulder function.

	Baseline	Post-Intervention	Within-Group Mean Changes	*d*	Between-Groups Mean Changes
Upper horizontal distance (mm)					
Group 1 (SRE)	87.2 (12.37)	82.4 (14.04)	4.8 [0.8/8.6] *	0.18	19.3 [9.7/28.7] ^††^
Group 2 (SRE + NMES)	86.2 (14.77)	63.1 (12.22)	23.1 [17.2/28.9] **	0.65	
Lower horizontal distance (mm)					
Group 1 (SRE)	89.7 (11.89)	79.9 (8.92)	9.8 [5.6/13.8] **	0.42	10.5 [4.3/16.8] ^†^
Group 2 (SRE + NMES)	88.4 (9.88)	69.4 (8.34)	19.0 [15.3/22.7] **	0.72	
IR RoM (°)					
Group 1 (SRE)	47.2 (3.68)	47.4 (2.31)	−0.2 [−2.0/1.7]		3.4 [0.6/7.5]
Group 2 (SRE + NMES)	45.2 (6.83)	44.0 (7.58)	1.2 [−1.7/4.1]		
ER strength (N)					
Group 1 (SRE)	44.15 (7.75)	49.05 (8.34)	−4.91 [−7.85/−1.96] *	0.29	10.79 [4.91/15.70] ^†^
Group 2 (SRE + NMES)	46.11 (7.26)	59.84 (6.67)	−13.73 [−17.66/−10.79] **	0.71	
ER strength (BW%)					
Group 1 (SRE)	7.67 (1.46)	8.47 (1.52)	−0.80 [−1.2/−0.3] *	0.26	1.45 [0.5/2.4] ^†^
Group 2 (SRE + NMES)	7.75 (1.16)	9.92 (1.15)	−2.17 [−2.72/−1.62] **	0.69	

SRE: scapular retraction exercise; NMES: Neuromuscular Electrical Stimulation; RoM: range of movement; N: newtons; BW %: body weight percent. Data are reported as mean (SD) or [95% confidence level]. * Indicates statistically significant within-group differences (*p* < 0.05). ** Indicates statistically significant within-group differences (*p* < 0.001). ^†^ Indicates statistically significant between-group differences (*p* < 0.05). ^††^ Indicates statistically significant between-group differences (*p* < 0.001). d = Cohen´s d.

## Data Availability

The data presented in this study are available upon request from the corresponding author.
